# Treatment of metastatic placental site trophoblastic tumor with surgery, chemotherapy, immunotherapy and coil embolization of multiple pulmonary arteriovenous fistulate

**DOI:** 10.1016/j.gore.2021.100782

**Published:** 2021-05-07

**Authors:** A. Porter, J.M. Barcelon, R.L. Budker, L. Marsh, J.M. Moriarty, X. Aguiar, J. Rao, E. Ghorani, B. Kaur, G. Maher, M.J. Seckl, G.E. Konecny, J.G. Cohen

**Affiliations:** a University of California Los Angeles, Division of Hematology Oncology, Los Angeles, CA, USA; b University of California Los Angeles, Division of Gynecologic Oncology, Los Angeles, CA, USA; c University of California Los Angeles, Division of Interventional Radiology, Los Angeles, CA, USA; d California Los Angeles, Department of Pathology, Los Angeles, CA, USA; e Gestational Trophoblastic Disease Centre, Charing Cross Hospital Campus of Imperial College London, United Kingdom

**Keywords:** Placental site trophoblastic tumor, Immunotherapy, Pembrolizumab

## Abstract

Placental Site Trophoblastic Tumor (PSTT) is a rare malignancy that often presents with extensive disease and can be resistant to traditional treatments. We present the case of a woman with stage IV PSTT who was initially managed with neoadjuvant chemotherapy followed by tumor debulking. Adjuvant therapy was guided by further pathologic analysis that revealed high levels of staining for PD-L1 as well as the presence of tumor infiltrating lymphocytes (TILs). Subsequently, the patient was treated with traditional chemotherapy with the EP/EMA regimen with the addition of pembrolizumab. The patient’s treatment course was complicated by the development of pulmonary arteriovenous malformations, autoimmune thyroiditis thought to be secondary to immunotherapy, and significant tinnitus secondary to platinum agents. Currently the patient is in follow up and remains in a complete remission.

## Highlights

• Placental site trophoblastic tumor can be resistant to chemotherapy.
• Multidisciplinary care is required for management of advanced disease.
• Increased PD-L1 expression can help guide use of immunotherapies.
• Complete responses are possible with aggressive multidisciplinary management.

## 1 Introduction

PSTT is a rare form of gestational trophoblastic neoplasia (GTN) and accounts for just 0.2% of all gestational trophoblastic disease. PSTT differs from other forms of GTN as they are slower growing, produce less hCG for the volume of disease present, metastasize later and involve lymph nodes more frequently (Schmid et al., 2009). As the disease is less chemosensitive, the use of single-agent chemotherapy is not appropriate and the International Federation of Gynecology and Obstetrics (FIGO) scoring system is not used. Surgery has an important role in the management of localized disease. Patients with disease localized to the uterus or with minimal metastatic disease should be counseled regarding hysterectomy because of the relative resistance of PSTT to chemotherapy. Stage-adapted treatment is determined by two independent poor prognostic factors: an interval of >48 months from the causative pregnancy and stage IV disease (Schmid et al., 2009, Froeling et al., 2019). Those with stage IV versus stage I disease irrespective of interval have an overall survival (OS) of 42% and 89%, respectively (Froeling et al., 2019).

To enable appropriately selected risk-adjusted treatment it is essential to have both a histological diagnosis providing material for genetic analysis to determine the causative pregnancy and whole-body imaging to assess spread of disease. Stage II-IV patients should be offered aggressive platinum-based chemotherapy including the option for experimental treatments such as high dose chemotherapy. More recently, immunotherapy may represent a promising treatment option for metastatic disease. Although PSTTs may not express program death-ligand 1 (PD-L1) as strongly as choriocarcinomas (Veras et al., 2017), success with metastatic PSTT has been reported with anti-PD1 treatment (Ghorani et al., 2017) and this is already playing an increasing role in the management of relapsed chemo-resistant tumors. Here we describe a clinical case of a patient with stage IV PSTT metastatic to the adrenal gland and lungs successfully treated with multimodal therapy: surgical debulking, combination chemotherapy and immunotherapy, and embolization of post treatment arteriovenous fistulae (pAVF). Immunotherapy was employed after discovery of elevated PD-L1 expression and significant involvement of tumor infiltrating lymphocytes (TILs).

## 2 Case report

A 34-year-old female, gravida 2, para 1, presented with a protruding vaginal mass 8 months following a preterm cesarean delivery at 27 weeks gestational age for preeclampsia and HELLP syndrome. A transvaginal ultrasound demonstrated an enlarged uterus, dilated varicose vessels throughout the myometrium with vascular structures extending to the vagina. MRI and CT scans confirmed vaginal involvement as well as enhancing left renal and right adrenal lesions, and multiple nodules in the lung bases were identified. The lung lesions measured up to 14 mm and some were in close proximity to the pulmonary veins concerning for vascular invasion. A brain MRI was unremarkable and a fluoroscopy-guided lumbar puncture was negative for malignant cells. The hCG levels of the spinal fluid were within the normal range; 3 IU/L (CSF/Serum ratio < 1:60). A PET/CT confirmed the aforementioned lesions albeit without significant FDG activity. A biopsy of the vaginal tumor was consistent with PSTT.

In an attempt to reduce the risk of pulmonary hemorrhage from high burden of disease the patient initially received treatment with low dose etoposide (E) 100 mg/m^2^ and cisplatin (P) 20 mg/m^2^ on days 1 and 2, repeated every week for 3 weeks. Five weeks after initiation of low dose EP the hCG level stabilized at 1138 from 1114 mIU/mL. Given the significant tumor burden in the uterus, the decision was made to proceed with cytoreductive surgery with hysterectomy. Preoperative imaging confirmed there was significant collateral vascularity involving the primary tumor. The patient underwent bilateral uterine artery embolization in order to reduce risk of hemorrhage prior to the planned surgery which included an exploratory laparotomy, total abdominal hysterectomy, bilateral salpingectomy, and excision of the vaginal tumor. Postoperatively the hCG level decreased to 472 mIU/mL.

**Fig. 1:**
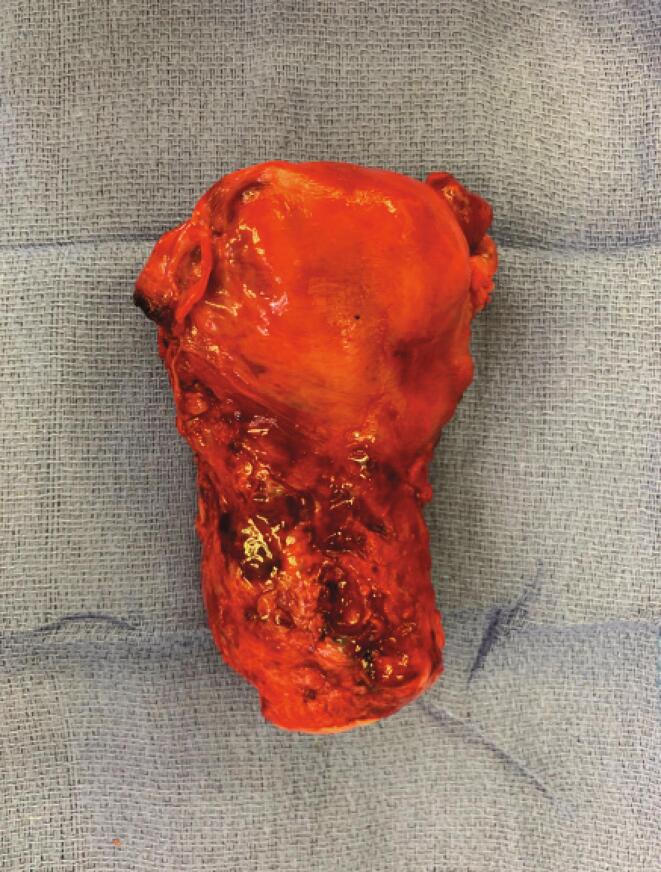
Image of uterus demonstrating vascularity involving the lower uterine segment.

**Fig. 2:**
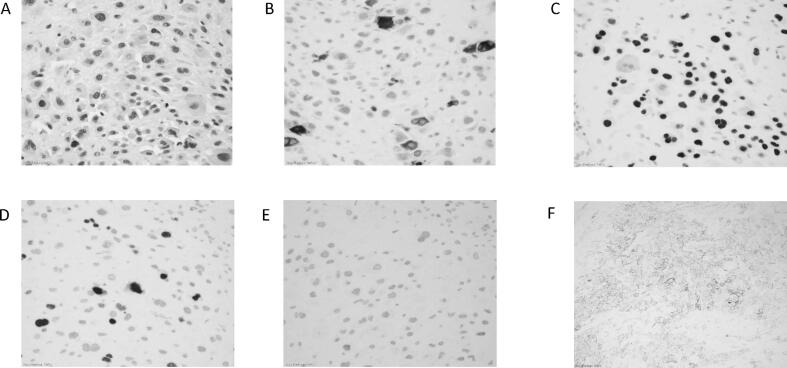
IHC stains A. H&E (40×) B. HCG (40×) C. GATA3 (40×) D. Ki67 (40×) E. P63 (40×) F. PDL1 (20×).

Gross surgical pathology revealed tumor infiltrating the entire uterine wall to the serosal surface with parametrial margin involvement, anterior vaginal wall involvement and lymphovascular space involvement (Fig. 1). Immunohistochemical (IHC) analysis (Fig. 2A) shows tumor cells with large pleomorphic nuclei and abundant eosinophilic cytoplasm. There was scattered expression of HCG (Fig. 2B) and diffuse positive staining for GATA3 (Fig. 2C). Ki67 staining (Fig. 2D) revealed a mildly increased proliferation index of 15–20%. There was no expression of p63 (Fig. 2E). PD-L1 was found to be highly expressed (90% (in house mAB) and 100% using the Tumor Proportion Score (TPS; Dako 22C3 pharmDx™) (Fig. 2F).

Post operatively the EP/EMA (etoposide, cisplatin/etoposide, methotrexate, actinomycin D) regimen was initiated intravenously: day 1 cisplatin 75 mg/m^2^ and etoposide 150 mg/m^2^, day 8 actinomycin D 0.5 mg, day 10 etoposide 100 mg/m^2^ and methotrexate 300 mg/m^2^. With completion of 5 cycles of EP/EMA, the hCG had decreased from 472 to 55 mIU/mL.

After cycle 5, the patient developed left sided upper abdominal pain and a follow up PET/CT demonstrated bilateral pulmonary nodules, some increased and some decreased since the prior exam. In addition, a splenic vein thrombosis with splenic infarct involving the middle and lower pole of the spleen was noticed for which she was started on anticoagulation with apixaban. Two weeks after completion of cycle 5 she developed chills and fever (38.5 °C). Blood cultures revealed methicillin-sensitive staphylococcus (MSSA) bacteremia thought to be secondary to port-a-cath infection. A transthoracic echocardiogram revealed a 2.5 cm right atrial mass (thrombus versus vegetation) as well as a patent foramen ovale. An MRI of the brain revealed multiple embolic infarcts. On additional review of the imaging by interventional radiology (IR), it was determined the patient had multiple pulmonary arterial pseudo-aneurysms with arteriovenous shunting. These correlated with the sites of previous lung nodules and were thought to be the result of vascular trauma due to regression of prior PSTT metastases.

**Fig. 3:**
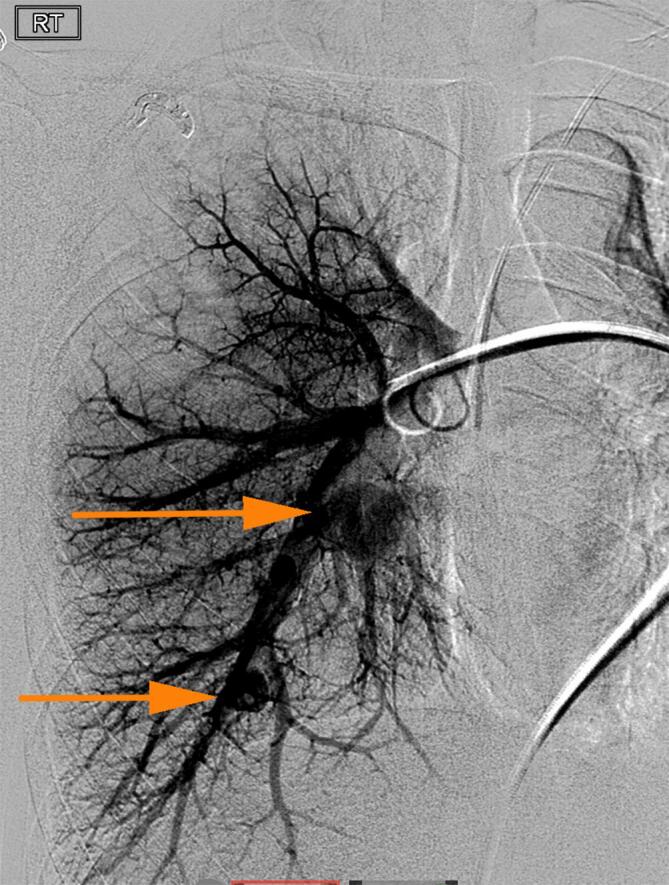

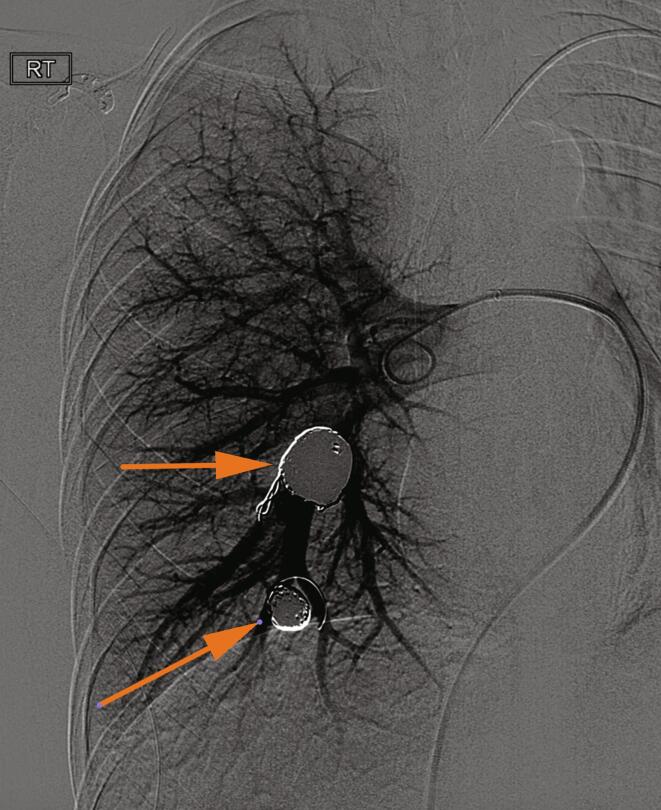
(a) Digital subtraction angiogram of the right pulmonary arteries demonstrates two large pseudoaneurysms with fistulous communication to the pulmonary vein. (b) Following coil embolization there is no residual shunting and no further right sided pulmonary arterial aneurysms or pseudoaneurysms.

In order to mitigate the risk of paradoxical embolus as well as pulmonary hemorrhage, she underwent transarterial embolization of these pulmonary shunts. A large arterial aneurysm within the right lower lobe of the lung as well as a large right lower lobe arteriovenous fistula with associated aneurysm were successfully treated with coil embolization (Fig. 3). The patient was bridged from intravenous heparin to apixaban on discharge and completed a four-week course of intravenous oxacillin. An interval MRI performed 1 month later revealed resolution of right atrial thrombus, and improvement in cystic lung disease, with a newly identified small pseudoaneurysm in the left lower lobe. The patient again underwent successful IR coil embolization of this lesion with subsequent resolution.

**Fig. 4:**
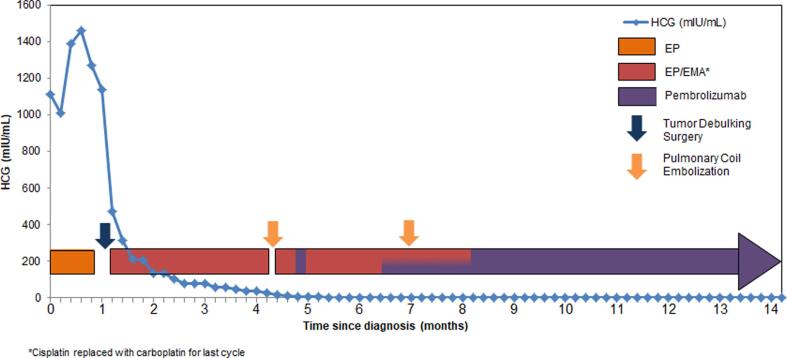
Chemotherapy and immunotherapy treatment cycles with correlating HCG levels. Surgery took place five weeks after initiation of EP with a total of 3 weeks of treatment given prior to hysterectomy.

Given the high PD-L1 expression of the tumor as well as the presence of TILs (40% and 10% positive CD4 and CD8 cells, respectively) the decision was made to add pembrolizumab 200 mg every three weeks starting with cycle 6. At the time of the third pembrolizumab infusion, the patient’s hCG level became undetectable. Chemotherapy with EP/EMA in addition to pembrolizumab was continued for an additional 2 months thereafter as consolidative therapy before transitioning to maintenance pembrolizumab (Fig. 4). The patient experienced several toxicities secondary to chemotherapy including thrombocytopenia as well as ototoxicity and tinnitus that was confirmed with formal audiological evaluation. This prompted the substitution of carboplatin for cisplatin with cycle 10 in the hopes of minimizing further adverse events.

Several days after the patient’s second infusion of pembrolizumab the patient developed symptomatic palpitations with heart rates elevated to the 130–140 range. Thyroid function tests (TFTs) were significantly elevated with a free T4 of 1.3 ng/dL, TSH of 8.2 mIU/mL, free T3 of 122 pg/dL, and total T3 of 36 ng/dL most consistent with inflammatory thyroiditis secondary to pembrolizumab. The patient required administration of methimazole and beta blockade followed by thyroid supplementation with resolution of TFT abnormalities as well as symptoms. The patient completed a total of 10 cycles of adjuvant chemotherapy with an end of treatment PET/CT demonstrating continued treatment response with an undetectable HCG level. The patient remains on pembrolizumab and disease free 7 months since completion of chemotherapy.

To enable appropriate counseling on the future prognosis, genetic analysis was performed to determine the causative pregnancy. DNA from tumor specimens, maternal and paternal tissue (blood), as well as child (saliva) was evaluated for 24 short tandem repeat loci as previously described at the Gestational Trophoblastic Disease Service at the Imperial College Hospital in London, UK (Zhao et al., 2016). Because all of the informative paternal alleles present in the tumor were also present in the female child born 8 months prior to the PSTT diagnosis, the antecedent pregnancy was found to be the source of the tumor.

## 3 Discussion

Here we describe the case of a patient with diffusely metastatic PSTT successfully treated with multimodal therapy including surgical debulking, combination chemotherapy and immunotherapy, and IR embolization of post treatment arteriovenous fistulae. Generally, definitive surgical management is reserved for those with early-stage disease, however given this patient’s largest volume of tumor was involving the uterus, the decision was made to proceed with surgery in order to achieve local control after EP had been given to minimize risk of bleeding from lung metastases. Review of the literature demonstrates improvement in long term disease control with combination of surgery and systemic treatment with advanced stage disease (Gadducci et al., 2019).

The use of immunotherapy was exquisitely effective in the management of this patient. GTN (invasive mole and choriocarcinoma) strongly expresses PD-L1 (Veras et al., 2017) and therefore the successful use of checkpoint inhibitors has increased. Pembrolizumab (anti-PD-1) has induced complete responses in 75–80% of unresectable, chemo-resistant GTN including cases that had failed high dose chemotherarpy (Ghorani et al., 2017, Clair et al., 2020, Huang et al., 2017). Currently, it is unclear how to select responding cases as all are PD-L1 positive and mutational burden may be absent or extremely low in GTN. Detection of TILs and HLA-G expression may be important but much more work is required. The PD-L1 inhibitor avelumab has also shown efficacy in GTN, inducing complete serological response in approximately 53% of patients who had previously been treated with single agent chemotherapy (You et al., 2020).

It is well known that GTN can have the propensity to form uterine AVMs, however the development of AVMs in the lung following treatment effect in pulmonary metastasis is rare. Patients can experience significant complications as a result of these AVMs if not promptly managed including hemorrhage and paradoxical brain emboli (Yamanari et al., 2017). Thankfully, these shunts or post-treatment fistulae are able to be definitively managed either with coiling via interventional radiology, as in our case, or with surgery. A multidisciplinary approach to the management of patients with metastatic PSTT is imperative. In this case, gynecologic oncology, medical oncology, interventional radiology, pathology, and cardiology experts were imperative to maximize this young woman’s cancer care. A combination of surgery and systemic treatment with the growing potential of immunotherapy should be considered in the setting of metastatic PSTT.

## Declaration of Competing Interest

The authors declare that they have no known competing financial interests or personal relationships that could have appeared to influence the work reported in this paper.
